# A New Technique Using a 4-0 Nylon Thread as a Guide for Easy and Precise Tube Insertion of Ahmed Glaucoma Valve Implant Into Ciliary Sulcus

**DOI:** 10.7759/cureus.34854

**Published:** 2023-02-11

**Authors:** Keisuke Nitta, Hideo Akiyama

**Affiliations:** 1 Department of Ophthalmology, Gunma University Graduate School of Medicine, Maebashi, JPN

**Keywords:** intraocular lens oscillation, trauma, glaucoma, case report, poor mydriasis, tube implant, nylon, ahmed glaucoma valve, ciliary sulcus

## Abstract

We present a new technique for inserting the tube of the Ahmed glaucoma valve (AGV) (model FP7; Rancho Cucamonga, CA: New World Medical) implant into the ciliary sulcus, easily and precisely, using a 4-0 nylon thread as a guide. An 88-year-old woman received AGV implantation for secondary angle recession glaucoma with underlying pseudoexfoliation syndrome in her left eye. She had a history of trauma with mild intraocular lens (IOL) oscillation and poor mydriasis, with maximum pupil diameter of 3.5 mm. Ciliary sulcus tube insertion in such patients sometimes becomes difficult, however, using a 4-0 nylon thread as a guide, precise insertion was achieved easily in the following way. A 4-0 nylon thread was placed into the anterior chamber through a 1 mm incision opposite the site of the AGV implant. Subsequently, a 23G needle was inserted into the sclera 2 mm from the corneal limbus in the same quadrant as AGV implants. The tip of the 23G needle proceeded horizontally to the iris, through the sclera and ciliary body, and into the ciliary sulcus. At the center of the pupil, the 4-0 nylon thread was introduced into the lumen of the 23G needle. Subsequently, the 23G needle, together with the 4-0 nylon thread in the lumen was withdrawn out of the eye. The 4-0 nylon was then inserted into the tube lumen of the AGV implant. Finally, by using 4-0 nylon as a guide, the Ahmed tube was inserted into the ciliary sulcus precisely without much difficulty.

## Introduction

Ciliary sulcus tube insertion of Ahmed glaucoma valve (AGV) implant is a useful method nowadays as it is less likely to cause corneal endothelial cell loss than anterior chamber tube insertion [1,2]. However, the low rigidity of AGV tube causes difficulties in insertion to ciliary sulcus, such as straying into the vitreous cavity or into an unidentifiable position, when using simple insertion methods [3]. Several techniques have been reported to overcome these problems, although problems remain, such as the complexity and unreliability of the procedure, and costs of the materials used [4-8]. In this study, we report a simple and precise technique of AGV tube insertion into the ciliary sulcus using inexpensive 4-0 nylon thread as a guide.

## Case presentation

An 88-year-old woman, with underlying both eyes pseudophakic and pseudoexfoliation syndrome, presented to us with uncontrolled intraocular pressure in the left eye for further management. She had undergone cataract surgery in both eyes 22 years ago, and since then had visited her local ophthalmologist for pseudoexfoliation (PEX) in her left eye, with intraocular pressure in the normal range. One year and six months ago, she sustained a fall and had a left eye traumatic infraorbital wall fracture with periorbital bruises. One year ago, the intraocular pressure (IOP) in her left eye increased to 43 mmHg, and she had been followed up with increased eye drops. The IOP gradually became uncontrolled, and she was referred to our department for surgery. At initial visit, corrected visual acuity was 20/25 in the right eye and 20/50 in the left eye, and IOP was 22 mmHg in the right eye and 40 mmHg in the left eye. Corneal endothelial cell density was 2577 cells/mm^2^ in the right eye and 2213 cells/mm^2^ in the left eye. A prostaglandin analog and a carbonic anhydrase inhibitor/α2-adrenergic receptor agonist eye drops were used in her left eye. Gonioscopic examination revealed angle recession from 2 to 7 o'clock in her left eye. In addition, her left eye showed poor mydriasis due to PEX, with a maximum pupil diameter of about 3.5 mm and mild intraocular lens oscillation.

**Figure 1:**
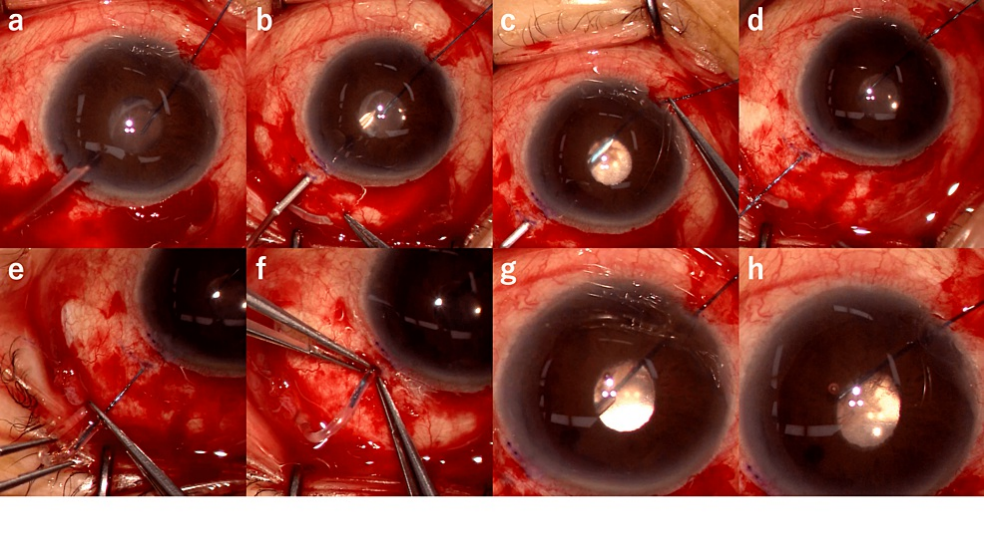
Intraoperative images of 4-0 nylon guided tube insertion into ciliary sulcus from the surgeon’s view. (a) A 4-0 nylon thread was inserted into anterior chamber from 1 mm incision. Poor mydriasis was seen. Before the process, the anterior chamber had been replaced with viscoelastic material, and the ciliary sulcus was also dilated. (b) A 23G needle was inserted into the sclera 2 mm from the corneal limbus, and the tip of the needle was advanced through the sclera and ciliary body, and into the ciliary sulcus, parallel to the iris plane. The tip of the needle was seen at the center of the pupil. (c) At the center of the pupil, the 4-0 nylon thread was introduced into the lumen of the 23G needle using forceps outside the eye. (d) The 23G needle was withdrawn out of the eye so that the 4-0 nylon in the lumen was also withdrawn out of the eye. (e) The 4-0 nylon was inserted into the tube lumen of the AGV implant using forceps. (f) The tube and the 4-0 nylon were inserted into the eye by forceps. (g) With the 4-0 nylon as a guide, the tube tip was precisely inserted through the sclera and ciliary body and into the ciliary sulcus. The tip of the tube was visible through the pupillary margin. (h) The 4-0 nylon was withdrawn out of the tube lumen, and the tube was left precisely into the ciliary sulcus.

**Figure 2:**
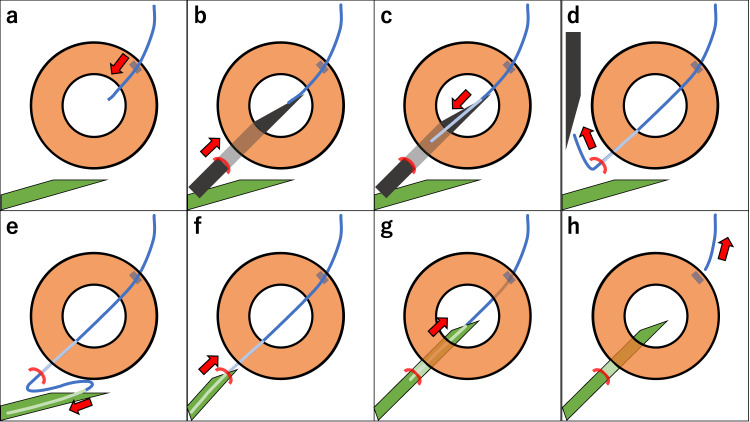
Schematic illustration of 4-0 nylon guided tube insertion into ciliary sulcus from the surgeon’s view. The direction and focus of each step are illustrated by the red arrows. Blue line indicates a 4-0 nylon thread. Black trapezoid indicates a 23G needle. Green trapezoid indicates the tube of AGV implant. Blue square indicates a corneal 1 mm incision. Red curve indicates a scleral insertion 2 mm from corneal limbus. (a) A 4-0 nylon thread is inserted into anterior chamber from 1 mm incision. (b) A 23G needle is inserted into the sclera 2 mm from the corneal limbus, and the tip of the needle proceeded horizontally to the iris until the tip can be seen through the pupil. (c) At the center of the pupil, the 4-0 nylon thread is introduced into the lumen of the 23G needle. (d) The 23G needle is withdrawn out of the eye so that the 4-0 nylon in the lumen is also withdrawn out of the eye. (e) The 4-0 nylon is inserted into the tube lumen of the AGV implant. (f) The tube and the 4-0 nylon are pushed into the eye. (g) With the 4-0 nylon as a guide, the tube tip is precisely implanted through the sclera and ciliary body and into the ciliary sulcus. (h) The 4-0 nylon is withdrawn out of the eye from the 1 mm incision. The image is created by the authors of this study.

She has proceeded with AGV implant surgery in her left eye as the following procedure. Conjunctival incision in the superior temporal quadrant followed by sub-Tenon's capsule anesthesia was performed. Insertion sites of the superior rectus and external rectus muscles were identified, and the plate of the AGV implant was inserted into the space and fixed with 7-0 nylon threads at a position 9 mm from the corneal limbus. The tube was trimmed to length with the tip visible from the pupil. A 1 mm incision was made in the inferior nasal cornea and a viscoelastic material was injected into the anterior chamber to dilate the ciliary sulcus. Then, a 4-0 nylon thread was placed into the anterior chamber through a 1 mm incision (Figures 1a, 2a). Subsequently, a 23G needle was inserted into the sclera 2 mm from the corneal limbus and the tip of the needle was advanced through the sclera and ciliary body, and into the ciliary sulcus, parallel to the iris plane (Figures 1b, 2b). At the center of the pupil, the 4-0 nylon thread was introduced into the lumen of the 23G needle (Figures 1c, 2c). The 23G needle was then withdrawn out of the eye so that the 4-0 nylon in the lumen was also withdrawn out of the eye (Figures 1d, 2d). The 4-0 nylon was then inserted into the tube lumen of the AGV implant (Figures 1e, 2e). Pushing the tube and the 4-0 nylon into the eye (Figures 1f, 2f), with the 4-0 nylon as a guide, the tube tip was precisely implanted through the sclera and ciliary body and into the ciliary sulcus (Figures 1g, 2g). Finally, the 4-0 nylon was withdrawn out of the eye from the 1 mm incision (Figures 1h, 2h). The tube was covered with preserved sclera, and the conjunctiva was closed. At one month postoperatively, the tube was positioned appropriately in the ciliary sulcus without any interference to the iris. The IOP was 10 mmHg without glaucoma eyedrops, and the corneal endothelial cell density was 2545 cells/mm^2^ with no decrease compared to the preoperative density.

## Discussion

Ciliary sulcus insertion is superior to anterior chamber insertion when compared with the corneal endothelial cell density loss rate [1,2]. However, ciliary sulcus insertion is more difficult than anterior chamber insertion because the tube passes through invisible parts, such as the ciliary body and the back surface of the iris. Further, weak zonules, the formation of Elschnig pearls, or poor dilation of the pupil make ciliary sulcus insertion difficult [5]. Asaoka et al. reported that 42/91 eyes (46%) were not successfully placed to ciliary sulcus with a single attempt, and 4/91 eyes (4.4%) were unable to achieve ciliary sulcus insertion [3]. A technique for ciliary sulcus insertion has been reported using 10-0 nylon thread with straight needle to create a sliding knot, but the technique is complicated [4].

Kasuga et al. have reported a tube insertion method using 4-0 nylon threads such as ours [5]. In their method, a shortcut 4-0 nylon thread is placed in the tube lumen as a stent outside the eye to increase rigidity and facilitate insertion to the ciliary sulcus. However, this method has a potential risk of straying into the wrong place, such as the vitreous cavity, as it passes blindly through the sclera and ciliary body without a precise guide. In fact, the method of Kasuga et al. is reported to be a proline-assisted ciliary sulcus tube insertion method. On the other hand, our technique uses a 4-0 nylon thread as a guide, which completely passes through the same way as the 23G needle, so that no risk of straying is present. Furthermore, the method of Kasuga et al. required supplies such as disposable forceps to remove the proline from the anterior chamber, whereas in our method, the 4-0 proline thread can be removed from the anterior chamber by simply pulling it out of the eye and no forceps are required [5].

In recent years, methods using a 21G needle [7,8] or a 23G needle as a guide have also been reported [6]. In these methods, the 21G or 23G needle penetrates in a straight direction with an incline from the corneal wound 180° opposite to the AGV implant to the sclera at the tube insertion site. Therefore, the tube inserted using that needle as a guide is also inclined and at risk of tilting towards the iris and interfering with the iris back surface. On the other hand, our technique has lower risk of interfering with the iris because the 23G needle is inserted into the eye horizontally to the iris. In addition, compared to our technique, the method using a 23G needle requires a 9-0 or 10-0 nylon thread with needle [6], thus using more supplies, while the method using a 21G needle has the disadvantage that the wound is larger [7,8].

A 4-0 nylon thread occupies 62% of the AGV tube lumen and is placed into the tube lumen after AGV surgery for hypotony [9]. Although a 5-0 nylon thread or a 3-0 nylon thread can also enter the tube lumen, a 4-0 nylon thread seemed to be the appropriate size, thus used in this case. Since this is a case report, further studies are required in a large number of cases and the thread size needs to be considered.

## Conclusions

Although tube insertion into the ciliary sulcus of AGV implants is a useful method, simple insertion methods sometimes have difficulty inserting the tube into the ciliary sulcus, resulting in straying into the vitreous cavity or insertion into unidentifiable positions. Our technique achieved simple and precise insertion of an AGV tube into the ciliary sulcus using inexpensive 4-0 nylon thread as a guide in a patient with IOL oscillation and poor mydriasis, who would otherwise have had difficulty with tube implantation into the ciliary sulcus.
